# Energy disruptors: rising stars in anticancer therapy?

**DOI:** 10.1038/oncsis.2015.46

**Published:** 2016-01-18

**Authors:** F Bost, A-G Decoux-Poullot, J F Tanti, S Clavel

**Affiliations:** 1 INSERM, C3M, U1065, Team Cellular and Molecular Physiopathology of Obesity and Diabetes, Nice, France; 2 Univ. Nice Sophia Antipolis, C3M, U1065, Nice, France

## Abstract

The metabolic features of tumor cells diverge from those of normal cells. Otto Warburg was the first to observe that cancer cells dramatically increase their glucose consumption to generate ATP. He also claimed that cancer cells do not have functional mitochondria or oxidative phosphorylation (OXPHOS) but simply rely on glycolysis to provide ATP to the cell, even in the presence of oxygen (aerobic glycolysis). Several studies have revisited this observation and demonstrated that most cancer cells contain metabolically efficient mitochondria. Indeed, to sustain high proliferation rates, cancer cells require functional mitochondria to provide ATP and intermediate metabolites, such as citrate and cofactors, for anabolic reactions. This difference in metabolism between normal and tumors cells causes the latter to be more sensitive to agents that can disrupt energy homeostasis. In this review, we focus on energy disruptors, such as biguanides, 2-deoxyglucose and 5-aminoimidazole-4-carboxamide ribonucleotide, that interfere with the main metabolic pathways of the cells, OXPHOS, glycolysis and glutamine metabolism. We discuss the preclinical data and the mechanisms of action of these disruptors at the cellular and molecular levels. Finally, we consider whether these drugs can reasonably contribute to the antitumoral therapeutic arsenal in the future.

## Introduction

Cancer cells are characterized by uncontrolled and rapid proliferation. Deregulation of the cell division machinery requires metabolic adjustments to provide macromolecules and energy to fuel cell growth and division. In the presence of oxygen, glucose is converted via glycolysis into pyruvate, which is then transported to the mitochondria to be transformed into acetyl-CoA by pyruvate dehydrogenase for integration into the tricarboxylic acid cycle (TCA). The TCA provides intermediates for biosynthetic reactions, citrate, aspartate and two essential cofactors for the electron transport chain: NADH and FADH_2_. Otto Warburg^1^ was the first to demonstrate that the metabolism of cancer cells differs from normal cells. Even in the presence of oxygen, cancer cells reprogram their utilization of glucose and favor the production of lactic acid instead of transporting pyruvate into the mitochondria. Although Warburg named this process ‘fermentation', the process is currently better known as ‘aerobic glycolysis'. This metabolic switch seems counterintuitive for rapidly dividing cells, which require large amounts of energy. Indeed, glycolysis is 18 times less efficient than mitochondrial oxidative phosphorylation for the production of ATP, and cells must adapt to compensate. To do so, the cells upregulate glucose uptake mainly via upregulating the expression of the glucose transporter Glut1. This avidity for glucose has proven useful for tumor detection and serves as a basis for detecting tumor cells by [^18^F] fluorodeoxyglucose positron emission tomography imaging.

**Figure 1:**
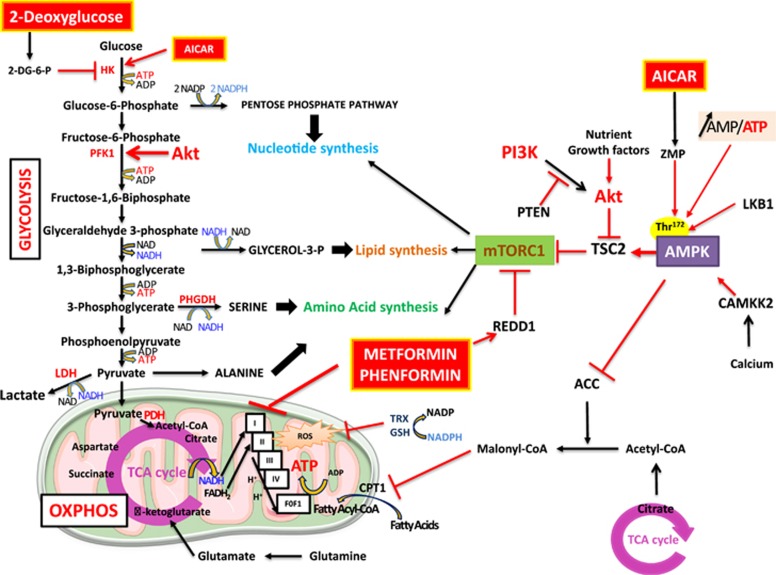
Molecular and cellular mode of action of energy disruptors. 2-Deoxyglucose inhibits glycolysis, it is phosphorylated by the hexokinase (HK) to produce 2-deoxglucose-6-phosphate (2-DG-6-P). Biguanides (metformin and phenformin) inhibit complex 1 of the electron transport chain (complexes 1 to 4 and the F0F1 ATP synthase). AICAR is converted in ZMP which activates the AMP-activated kinase (AMPK). The glycolysis converts glucose in pyruvate via a sequence of enzymatic reactions. The lactate dehydrogenase (LDH) catalyzes the conversion of pyruvate into lactate. Pyruvate can be addressed to the mitochondria and converted into acetyl-CoA by the pyruvate dehydrogenase (PDH), acetyl-CoA is then processed by the tricarboxylic acid (TCA) cycle. The TCA produces important intermediates but also cofactors (NADH and FADH_2_) required by the electron transport chain. The AMPK is phosphorylated by LKB1 or the CAMKK2 and activates TSC2 an inhibitor of mTORC1. It also inhibits and phosphorylates the acetyl-CoA carboxylase (ACC), the enzyme that converts the acetyl-CoA into malonyl-CoA, an inhibitor the Carnitine palmitoyltransferase-1 (CPT-1). Akt is activated by growth factors and PTEN is a negative regulator of the PI3K/Akt pathway. Akt phosphorylates and inhibits TSC2. CAMKK2, calcium/calmodulin-dependent protein kinase kinase 2; GSH, glutathione; PFK-1, phosphofructokinase-1; PHGDH, phosphoglycerate dehydrogenase; PI3K, phosphoinositide 3-kinase; TRX, thioredoxin; TSC2, tuberous sclerosis 2.

The reliance on glycolysis is associated with the activation of oncogenic pathways. One of the most commonly altered signaling pathway in human cancer is the phosphoinositide 3-kinase (PI3K) pathway. This pathway is activated in response to growth factors and by mutations in the tumor suppressor gene Phosphatase and TENsin homolog (PTEN). Once activated, the phosphoinositide 3-kinase pathway strongly promotes cancer cell proliferation and survival but also affects cell metabolism. The main effector of the phosphoinositide 3-kinase pathway is Akt. Akt is a regulator of glycolysis and plays a major role in the regulation of the bioenergetic balance. It stimulates glycolysis by increasing the expression and translocation of glucose transporters.^2^ In addition, Akt indirectly activates the rate-limiting enzyme of glycolysis, phosphofructokinase-1, by phosphorylating phosphofructokinase-2, which produces fructose-2,6-bisphosphate, the most potent activator of phosphofructokinase-1.^3^ Finally, Akt is a strong activator of the mechanistic target of Rapamycin (mTOR) by phosphorylating and inhibiting tuberous sclerosis 2, the negative regulator of mTOR. mTOR is an indispensable catalytic subunit of two distinct protein complexes, mTOR complex 1 (mTORC1) and mTOR complex 2. Both complexes are key metabolic and bioenergetic checkpoints that integrate growth signaling and nutrient availability.^4, 5^ Activated Akt strongly stimulates mTORC1, which positively regulates protein, lipid and nucleotide synthesis in response to sufficient nutrient and energy conditions^6^ (Figure 1). mTORC1 activation is a strong antiapoptotic and prosurvival signal.

When the nutrient supply is low, cells slow their metabolism to inhibit anabolic reactions and avoid energy shortage and death through the inhibition of mTORC1. The AMP-activated protein kinase (AMPK) is the main sensor of energy in cells. An increase in the AMP/ATP ratio induces the activation of AMPK.^7^ AMPK phosphorylates and activates tuberous sclerosis 2, the negative regulator of mTOR. This signal opposes the effects of Akt and acts as a potent inhibitor of mTORC1. AMPK is a major adaptive kinase and a heterotrimeric enzyme complex; it comprises one catalytic α-subunit and two regulatory β and γ subunits. Upon an energy stress, AMP and ADP bind to the γ-subunit and induce a conformational change that enhances its activity and the phosphorylation on Thr^172^ by the liver kinase B1 (LKB1). LKB1 is the upstream kinase required for the activation of AMPK in response to an energy stress. LKB1 is somatically mutated in some non-small-cell lung cancer and cervical carcinoma. Furthermore, germline mutations of LKB1 are responsible for the Peutz–Jeghers syndrome,^8^ which is characterized by the development of gastrointestinal and oral lesions. Importantly, AMPK can also be phosphorylated in response to calcium flux independently of LKB1 by the calcium/calmodulin-dependent protein kinase kinase 2. In addition, MAPKKK TAK1/MAP3K7 has been reported to phosphorylate Thr^172^ of AMPK.^9^

Considering the importance of the energy supply for cancer cells, the considerable interest in molecules interfering with the energy levels of cells is not surprising. Indeed, the induction of a strong energy stress in cancer cells leads to a major depletion of ATP, and consequently, to cell death. In this review, we will focus on three different promising molecules that interfere with energy cell metabolism: (i) biguanides (metformin and phenformin), used for the treatment of type II diabetes, (ii) 2-deoxyglucose, a potent inhibitor of glycolysis and (iii) Acadesine, also known as AICAR (5-aminoimidazole-4-carboxamide ribonucleotide), an energy restriction mimetic agent.

## The biguanides: mitochondrial disruptors

The most popular drugs among this family of anti-diabetic molecules are metformin and phenformin. Phenformin was withdrawn from the market due to its toxic effects, mainly lactic acidosis. Phenformin was replaced by metformin, which is now the first-line treatment for type II diabetes, with more than 120 million prescriptions worldwide. Metformin, which has been used for decades in the treatment of diabetes, offers an excellent side effect profile. It does not induce hypoglycemia but does provoke some gastrointestinal disorders (diarrhea, bloating and nausea), which can be partially prevented by a progressive increase in the dosage. The rare cases of lactic acidosis reported (<1/10 000) have occurred predominantly in patients with poor renal function. The mechanism of metformin action has been well studied in liver, adipose tissue, skeletal and heart muscles. The glucose-lowering effect is primarily a consequence of reduced hepatic glucose production, increased insulin sensitivity and glucose utilization by muscles and adipocytes, resulting in decreased insulinemia.^10^ Most of these effects are the result of the activation of AMPK by metformin.

### Biguanides regulate the AMPK/mTORC1 axis and reduce cancer incidence

The pioneering work of Zhou *et al.*^11^ in 2001 was the first demonstration that metformin induces the phosphorylation of AMPK on Thr^172^ (Figure 1). Then, the tumor suppressor LKB1 is required to phosphorylate AMPK in response to biguanides.^12^ The following question could then be asked: if metformin action requires LKB1 to activate AMPK, and AMPK inhibits mTORC1, what are the consequences in terms of cancer incidence in patients treated with metformin for decades? Evans *et al.*^13^ were the first to answer this question by demonstrating that metformin reduces the risk of cancer in a retrospective epidemiological study performed on 11 870 diabetic patients. Numerous retrospective studies followed, most of which examined specific cancers (breast, colon, pancreas and prostate) and drew the same conclusion. Of note, biases in some of the epidemiological studies have been reported, and the results should be considered with caution.^14^ Nevertheless, the promising results of the experimental studies warrant the numerous ongoing clinical trials of metformin in cancer therapy.

### Biguanides inhibit tumor growth and target cancer cell metabolism

Metformin and phenformin have a direct effect on tumor cells and inhibit the proliferation of numerous cancer cell lines.^15, 16^ They both inhibit tumor growth and metastasis formation in multiple animal models that develop spontaneous or engrafted tumors.^17, 18, 19, 20, 21^ At the cellular level, metformin induces apoptosis,^22^ cell cycle arrest via the downregulation of cyclin D1^15^ or autophagy,^23^ depending on the cancer cell line. However, the most striking discovery was certainly the effect of metformin on the energy metabolism of cancer cells. Two independent teams initially demonstrated that metformin inhibits complex 1 of the mitochondrial respiratory chain and targets oxidative phosphorylation (OXPHOS).^24, 25^ Both studies showed that biguanides decrease oxygen consumption and impact the mitochondrial membrane potential. This effect was attributed to a mild but significant inhibition of complex 1. Whereas one study showed that the effect of metformin requires intact cells,^24^ the other study demonstrated that the inhibitory effect occurs in isolated mitochondria.^25^ This discrepancy was attributed to the fact that the two studies were performed in two different mitochondrial respiratory states.^26^ The mode of action of biguanides on complex 1 remained unsolved until the recent work of Bridges *et al.* In this study, metformin and other biguanides were found to inhibit ubiquinone reduction, one of the multiple steps of the catalytic reaction of complex 1.^27^ The discovery that metformin inhibits complex 1 activity in cancer cells even more strongly than Rotenone, a classical inhibitor of complex 1, opened new horizons for biguanides in the field of cancer metabolism. The inhibition seems specific to cancer cells, but more importantly, induces a strong decrease in the intracellular ATP concentration.^28^ Are the antiproliferative effects of metformin due to the energy stress it generates? To answer this question, Wheaton *et al.* have overexpressed the *Saccharomyces cerevisiae* protein NDI1 in cancer cells. NDI1 is a single-subunit NADH dehydrogenase and is resistant to metformin. It oxidizes NADH in a process similar to that of the multi-subunit mammalian complex 1. The authors showed both *in vitro* and *in vivo* that overexpression of NDI1 reverses the antiproliferative and antitumoral action of metformin.^29^ As an energy disruptor, metformin has also been found to inhibit glucose production in hepatocytes^30^ and lipogenesis in prostate cancer.^31^ In response to the inhibition of complex 1 activity, cancer cells increase glucose consumption and glycolysis with an elevation of lactate production,^28^ decrease glucose oxidation and promote glutamine metabolism. Consequently, the inhibition of glutamine anaplerosis, a mechanism by which glutamine provides carbon to the TCA cycle via glutamate and α-ketoglutarate, by metformin further attenuates proliferation. Conversely, increasing glutamine metabolism rescues the antiproliferative effects of metformin.^32^ Together, these studies demonstrate that cancer cells activate compensatory pathways to counteract the metabolic chaos induced by metformin. Targeting these adaptations will improve the effect of biguanides and avoid resistance.

### Biguanides target cancer stem cells

Cancer stem cells (CSCs) are localized in tumors, resistant to chemotherapy, and capable of self-renewal and differentiation. Importantly, CSCs are the cause of disease relapse. Biguanides appear to target this cancer cell population. The combination of metformin with chemotherapy has been shown to be more efficient than either drug alone in xenograft models using several cancer cell lines, and this treatment specifically targets CSCs. Furthermore, treatment with both drugs significantly prolongs the remission following xenograft implantation.^33, 34^ This specific effect was confirmed in several other cancer models, including pancreas, breast and ovary.^35, 36, 37^ Interestingly, Sancho *et al.* have shown that CSCs rely mainly on OXPHOS and are unable to effectively induce glycolysis to compensate for reduced ATP production upon mitochondrial inhibition. The level of MYC expression controls this metabolic characteristic of CSCs; low MYC expression allows high PGC1-α expression, which results in enhanced mitochondrial biogenesis. Consequently, the observation that metformin specifically affects the viability of CSCs to a greater extent than non-CSCs is not surprising.^38^

### Biguanides and AMPK

Metformin and phenformin activate AMPK in most, if not all, cancer cells and consequently inhibit mTORC1 via tuberous sclerosis 2 protein. Several studies have shown that inhibition of AMPK with siRNA or compound C reverses the antiproliferative effects of metformin.^16, 39, 40^ However, we and others have found that metformin can mediate its effects independently of AMPK. We have shown that REDD1 (regulated in development and DNA damage responses 1) is upregulated in response to metformin and mediates its effects on mTORC1 and cell cycle arrest^41^ (Figure 1). Kalender *et al.*^42^ have demonstrated that metformin inhibits mTORC1 signaling in the absence of AMPK and tuberous sclerosis 1/2 and have shown that metformin affects mTORC1 via a Rag GTPase. Additional evidence for an AMPK-independent mechanism came from a study showing that metformin can still inhibit hepatic gluconeogenesis in mice lacking AMPK in the liver. According to Foretz *et al.*,^30^ this inhibition is also independent of LKB1. Again, these reports demonstrate the diversity of the cellular actions of metformin, and elucidating the metabolic actions of metformin will certainly be beneficial for the treatment of several human pathologies.

## 2-Deoxyglucose: the glycolytic disruptor

2-Deoxyglucose (2-DG), an inhibitor of hexokinase (HK), blocks the first and rate-limiting reaction of glycolysis and competitively inhibits glucose uptake. 2-DG is phosphorylated by HK to form 2-deoxy-d-glucose-6-phosphate (2-DG-6-P), which cannot be metabolized by glycolysis but accumulates and inhibits HK. Targeting glycolysis in cancer therapy is very pertinent due to several metabolic and biological effects. First, the inhibition of glycolysis decreases the production of glycolytic intermediates, such as glucose-6-P, glyceraldehyde-3-P and 3-phosphoglycerate, which are the precursors of nucleic acids, phospholipids and serine, respectively. Second, it decreases the antioxidant defenses of cancer cells. Third, it interferes with the *N*-glycosylation of proteins and induces endoplasmic reticulum (ER) stress. Finally, it induces an energy stress due to depletion of ATP.

### 2-DG interferes with anabolic reactions

The 2-DG-6-P produced by the phosphorylation of 2-DG by HK undergoes only the first enzymatic reaction in the pentose phosphate pathway to generate 2-DG-6-phosphogluconolactone, which cannot be further metabolized. The pentose phosphate pathway is a major metabolic pathway and is the principal source of NADPH and the source of ribose, the precursor of nucleotides. Glyceraldehyde-3-P is downstream of fructose 1,6-bisphosphate in glycolysis and generates glycerol-3-P, a precursor of phospholipids, which are the main components of the cellular membrane. Another important intermediate of glycolysis is 3-phosphoglycerate. 3-Phosphoglycerate is metabolized by 3-phosphoglycerate dehydrogenase, the first enzyme in the three-step serine biosynthetic pathway. 3-Phosphoglycerate dehydrogenase uses NAD as a cofactor to oxidize 3-phosphoglycerate into phosphohydroxypyruvate, which subsequent enzymes in the pathway convert into serine. Serine is essential for the synthesis of proteins and other biomolecules needed for cell proliferation, including nucleotides, phosphatidylserine and sphingosine. Overexpression of 3-phosphoglycerate dehydrogenase and increased biosynthesis of serine have been shown to be essential for the development of certain breast cancers.^43^ In conclusion, blocking glycolysis has a major impact on the principal anabolic pathways.

### 2-DG interferes with the antioxidant defenses

Reactive oxygen species (ROS) are primarily generated as by-products during mitochondrial electron transport. ROS cause numerous deleterious events, including inducing lipid peroxidation, DNA damage and protein oxidation. ROS thereby contribute to genomic mutations and an inability to differentiate and indirectly promote cell immortalization (which is a characteristic of cancer cells). Glycolysis, via the pentose phosphate pathway (which generates NADPH), attenuates the oxidative stress. NADPH provides reducing equivalents for glutathione- and thioredoxin-dependent peroxidase pathways,^44^ which are major antioxidants responsible for the detoxification of ROS and therefore protect against oxidative damage. Blocking glycolysis with 2-DG results in NADPH deficiency and induces the formation of ROS.^45, 46^ Indeed, only one of the two potential molecules of NADPH is produced by the pentose phosphate pathway when 2-DG is added to the cells. As reported earlier, 2-DG-6-phosphogluconolactone (the product of the conversion of 2-DG-6-P) cannot be further metabolized and therefore produces only one molecule of NADPH from NADP^+^. Treatment with 2-DG reduces the antioxidant potential of cancer cells and causes them to become more sensitive to oxidative stress induced by radiotherapy and chemotherapy.

### 2-DG interferes with the metabolism of glycoproteins

The glycosylation of proteins is an important biological process that promotes protein stability, proper protein folding and cell adhesion. Glycosylation consists of the addition of *N*-glycans to the nitrogen of asparagine or arginine of the proteins and occurs in the lumen of the ER. During the *N*-glycosylation reaction, mannose is converted to mannose-guanosine diphosphate (GDP), an important intermediate in glycan synthesis.^47^ Glucose participates in this process because glucose-6-P can be converted to mannose-6-phosphate and then to mannose-GDP. 2-DG is structurally similar to mannose and leads to the formation of 2-DG-GDP, which competes with mannose-GDP in the formation of oligosaccharide chains. Thus, the abnormal glycoproteins formed from 2-DG disrupt the *N*-glycosylation process and prevent normal protein folding. An accumulation of unfolded/misfolded proteins results in the activation of the unfolded-protein response, which acts to prevent ER stress. On the one hand, it inhibits protein synthesis, while on the other hand, it decreases the degradation of abnormal proteins. Treatment with 2-DG induces ER stress and the expression of ER-stress specific markers, including the C/EBP homologous (CHOP) protein, which plays an important role in ER-stress-induced cell death. Many studies suggest that the toxicity of 2-DG can be attributed to the inhibition of glycosylation. Indeed, addition of exogenous mannose can rescue cells from 2-DG-induced cell death but does not reverse the decrease in ATP.^48,49^

### 2-DG induces an energetic stress

The inhibition of glycolysis annihilates the two ATP-producing reactions in glycolysis: (1) the conversion of 1,3-bisphosphoglycerate into 3-phosphoglycerate, which generates two molecules of ATP from two molecules of ADP; and (2) the transformation of phosphoenolpyruvate into pyruvate by pyruvate kinase, which leads to the formation of two molecules of ATP. Ultimately, the net production of ATP by glycolysis is two molecules of ATP because HK and phosphofructokinase-1 both require one molecule of ATP. Importantly, blocking glycolysis decreases the concentration of pyruvate, the precursor of acetyl-CoA and citrate, which integrate into the TCA to ultimately produce ATP. Therefore, the inhibition of glycolysis affects OXPHOS via a reduction in TCA flux.

These actions lead to a decrease in intracellular ATP concentration, cell cycle blockade, inhibition of cell growth and cell death.^50, 51^

### The mimetic agent: AICAR

AICAR or Acadesine is a drug that directly activates AMPK. Inside the cells, AICAR is phosphorylated by adenosine kinase into 5-amino-4-imidazolecarboxamide ribotide (ZMP). ZMP is an analog of AMP and thus mimics several of its effects.^52^ Unlike biguanides and 2-DG, AICAR induces the phosphorylation and activation of AMPK but does not disturb the energy status of the cells. AICAR has been used for decades as an activator of AMPK and was recently used as a doping agent that increases running capacity without any training.^53, 54^

### AICAR interferes with biosynthetic pathways

AICAR has been shown to regulate several aspects of metabolism. Most of the pioneering studies on the activation of AMPK were performed using this drug in normal cells and insulin-sensitive organs. AICAR inhibits anabolic processes and promotes catabolic, ATP-generating reactions. For instance, AICAR inhibits lipid and cholesterol synthesis in the liver through the phosphorylation and inactivation of acetyl-CoA carboxylase and 3-hydroxy-3-methyl-glutaryl-CoA reductase (HMG-CoA reductase), respectively.^55^ On the other hand, a key biological effect of AICAR is the stimulation of fatty acid oxidation. AMPK phosphorylates and inactivates acetyl-CoA carboxylase, which catalyzes the formation of malonyl-CoA, an inhibitor of carnitine palmitoyl-transferase-1.^52^ Carnitine palmitoyl-transferase-1 is an enzyme associated with the outer membrane of the mitochondria that allows the entry of long-chain fatty acids into the mitochondria to be oxidized. AICAR is also a strong inhibitor of protein synthesis, again through AMPK, which phosphorylates and inactivates eukaryotic elongation factor 2 in hepatocytes.^56^ Numerous studies have also shown that AICAR inhibits the translation of proteins though its negative action on mTORC1. AICAR modulates the expression of transcription factors, such as SREBP1c and ChREBP, leading to the inhibition of lipogenesis. In skeletal muscle and heart, AICAR promotes glucose uptake and lipid oxidation. The stimulation of glucose uptake is attributed to an increase in glucose transporter expression and translocation to the membrane.^57^ Given the antihyperlipidemic and antihyperglycemic effects of AICAR and the anti-diabetic properties of the compound in mice,^58^ clinicians have started to use AICAR for the treatment of type II diabetes.^59, 60, 61^

### AICAR and cancer

Most of the metabolic effects described above provide evidence for a beneficial effect of AICAR in cancer cells. Indeed, numerous studies have shown that AICAR promotes cell cycle arrest and/or apoptosis in many cancer cell lines.^15, 62, 63, 64, 65, 66^ Interestingly, in some cases, the antitumoral effects of AICAR are independent of AMPK. Santidrian *et al.* have demonstrated that AICAR induces apoptosis in chronic lymphocytic leukemia, even when the catalytic unit of AMPK is deleted. Furthermore, the authors have shown that this proapoptotic effect is present in p53 mutated cells and is preceded by an increase in the proapoptotic proteins NOXA, BIM and PUMA.^67^ Recently, much attention has been paid to the effects of AICAR in hematological cancers, specifically in lymphoma, acute lymphoblastic leukemia and T-cell lymphoblastic lymphoma.^68, 69, 70^ In this model (as well as in other cancers, such as prostate cancer), AICAR is a much stronger activator of AMPK and inducer of apoptosis than biguanides or 2-DG.^15^ Notably, the concentration of AICAR required for the induction of apoptosis in cancer cells is within the millimolar range, which implies obvious problems for clinical use.

## The other side of AMPK activation

Although many studies support the tumor-suppressive role of AMPK, emerging evidence suggests that under certain circumstances, activation of AMPK protects against cell death. Indeed, activated AMPK confers metabolic adaptation to tumor cells which reduce anabolic reactions. Under nutrient starvation activated AMPK promotes cell survival and inhibits apoptosis.^71^ Not only AMPK activation maintains ATP levels but it also maintains NADPH levels which is crucial since it participates in redox regulation and cell survival. When activated, AMPK inhibits acetyl-CoA carboxylase and consequently the consumption of NADPH for fatty acid synthesis. In addition, it activates fatty acid oxidation which generates NADPH.^72^

Constitutive adaptation to energy stress has been shown in pancreatic cancer cells, and this tolerance to low glucose was attributable to the high expression level of AMPK. Knockdown of the catalytic unit of AMPK diminished the ability of cells to grow in glucose-free media.^73^ More recently, Park *et al.*^74^ showed that the downregulation of the AMPK α1 and α2 subunits inhibits prostate cancer cell growth and induced apoptosis. This other aspect of AMPK activation is important and requires attention when considering using AMPK activators for therapeutic use.

## What is the future for metabolic disruptors in the clinic?

### Metformin

The remarkable efficiency of biguanides to overcome cell and tumor proliferation in experimental studies and the evidence from epidemiological studies have prompted many clinicians to start clinical trials. Metformin can be prescribed by any physician and is safe and freely available. As a result of this excitement and easy access to the drug, more than 100 clinical studies have been launched (see clinicaltrial.gov). Under this unique scenario, some studies have not been as rigorous as other clinical trials using novel compounds. Therefore, when the results of the trials are available, we will need to carefully examine their design. Encouraging results concerning a small number of patients have recently been published. Hosono *et al.*^75^ have demonstrated that metformin (at the low 250 mg/day dosage for 1 month) decreases the number and size of colorectal aberrant crypt foci (an endoscopic surrogate marker of colorectal cancer) in non-diabetic patients. More recently, in patients with prostate cancer treated with metformin between the day of prognosis and the radical prostatectomy, Joshua *et al.*^76^ have observed a reduction in Ki67 staining and a significant decrease in the phosphorylation of P-4EBP1, a target of mTORC1. Conversely, the recent results of the first clinical trial performed on more than 100 patients with pancreatic cancer were disappointing. The addition of metformin at a conventional anti-diabetic dose (500 mg twice a day) to classical chemotherapy (erlotinib and gemcitabine) had no advantage for the survival of patients with advanced pancreatic cancers.^77^ Of note, pancreatic tumor cells mainly rely on glycolysis; therefore, metformin may not be a good candidate for this cancer. The coming year should be crucial to conclude whether metformin has a true beneficial effect in cancer.

### 2-DG

Based on Warburg's observation, 2-DG was investigated as a monotherapy in the 1950s, with no conclusive results.^78^ Because most cancer cells have normal capacities for using alternative sources of carbons through oxidative phosphorylation, this outcome is not surprising. More recent studies have tried to determine the tolerable and optimal dose of 2-DG for use in patients. Adverse side effects include fatigue, dizziness, restlessness and hypoglycemic symptoms. Depending on the study, the dose of 2-DG administered daily can vary from 45 to 300 mg/kg and is always combined with other anticancer treatments, such as radiotherapy or chemotherapy.^79, 80, 81^ Only one of these studies commented on the efficiency of 2-DG as an antitumoral agent. Indeed, Raez *et al.*^80^ found one patient with partial response after 1 week of 2-DG alone and 12 patients with stable disease among a total of 34 patients. Surprisingly, no other clinical trials using 2-DG are registered on the NIH clinical trial website.

### AICAR

Before the discovery of the action of AICAR on AMPK, this drug was described as a very potent cardioprotective agent with a different mechanism of action compared with standard nucleoside analogs, such as fludarabine.^82^ So far, only one clinical trial using AICAR in cancer has been reported in the literature, and few are mentioned on the cliniclatrial.gov website. The reported phase I/II clinical trial was performed in patients with B-cell chronic lymphocytic leukemia, which is the most frequent type of leukemia in the elderly in western countries. The symptoms of the disease arise from a clonal excess of B lymphocytes. *Ex vivo*, B cells have been found to be very sensitive to the proapoptotic effects of AICAR.^62^ A total of 24 patients were enrolled in the clinical trial, and AICAR had a very safe profile for doses between 50 and 250 mg/kg. Encouraging results were obtained; a reduction in palpable lymph nodes was observed in most patients.^83^

Interestingly, clinical trials combining two of these metabolic disruptors have not been performed. The combination of metformin, an inhibitor of OXPHOS, and 2-DG, the inhibitor of glycolysis, sounds promising. This approach relies on the induction of a major energetic stress targeting the two main ATP-producing pathways. *In vitro* and *in vivo* experiments have established the relevance of such a combination^84, 85^ with a strong reduction in tumor growth.

## Conclusion

Although the use of the metabolic disruptors alone is very efficient in isolated cancer cells, the use of these agents as monotherapy is certainly not warranted. Firstly, proposing a treatment with solely an anti-diabetic drug or a glucose analog or a doping agent to patients with advanced cancer is ethically questionable in a clinical trial. Secondly, the metabolic disruptors significantly improve the antitumoral effect of classical therapies, including chemotherapy and radiation, in the animal experiments. Thirdly, these metabolic drugs act as sensitizers and cause tumor cells to become more susceptible to a second hit.

In the future, better understanding the mode of action of these drugs not only in cancer cells but also on stromal cells will be important to overcome tumor heterogeneity and improve the efficiency of metabolic disruptors. Most laboratory *in vitro* studies use high concentrations of metformin, 2-DG and AICAR that are not compatible with the concentrations found in serum. Derivatives that improve the pharmacokinetics of these drugs are urgently needed. Metformin is highly polar and requires specific transporters to enter the cells, namely organic cation transporters. The responsiveness of tumor cells to metformin greatly depends on the expression of these organic cation transporters.^86^ In addition, Birsoy *et al.*^87^ have found that cell lines sensitive to low glucose are also defective in OXPHOS and that this traits confers sensitivity to biguanides. Thus, the expression of organic cation transporters and the metabolic characteristics of the cancer cells should be investigated and known prior to treatment with biguanides.

The tumor microenvironment plays a central role in the aggressiveness of tumors. The metabolic disruptors have been shown to interfere with stromal cell metabolism. For example, Beneteau *et al.* have elegantly demonstrated that 2-DG in combination with etoposide promotes an antitumoral immune response. In this study, only tumor cells treated with both agents ‘vaccinated' mice against tumor growth.^88^ More recently, metformin has been shown to increase CD8(+) tumor-infiltrating lymphocytes, which promote tumor rejection. Naive CD8^+^T lymphocytes treated with metformin migrate into the tumor and exert an antitumoral effect following adoptive transfer.^89^ A better understanding of the global effects of metabolic disruptors is needed, and the results from the ongoing clinical trials will certainly orientate the research in the field.

## References

[bib1] 1Warburg O. On the origin of cancer cells. Science 1956; 123: 309–314. 13298683 10.1126/science.123.3191.309

[bib2] 2Elstrom RL, Bauer DE, Buzzai M, Karnauskas R, Harris MH, Plas DR et al. Akt stimulates aerobic glycolysis in cancer cells. Cancer Res 2004; 64: 3892–3899. 15172999 10.1158/0008-5472.CAN-03-2904

[bib3] 3Deprez J, Vertommen D, Alessi DR, Hue L, Rider MH. Phosphorylation and activation of heart 6-phosphofructo-2-kinase by protein kinase B and other protein kinases of the insulin signaling cascades. J Biol Chem 1997; 272: 17269–17275. 9211863 10.1074/jbc.272.28.17269

[bib4] 4Rebsamen M, Pochini L, Stasyk T, de Araujo ME, Galluccio M, Kandasamy RK et al. SLC38A9 is a component of the lysosomal amino acid sensing machinery that controls mTORC1. Nature 2015; 519: 477–481. 25561175 10.1038/nature14107 PMC4376665

[bib5] 5Wang S, Tsun ZY, Wolfson RL, Shen K, Wyant GA, Plovanich ME et al. Metabolism. Lysosomal amino acid transporter SLC38A9 signals arginine sufficiency to mTORC1. Science 2015; 347: 188–194. 25567906 10.1126/science.1257132 PMC4295826

[bib6] 6Ben-Sahra I, Howell JJ, Asara JM, Manning BD. Stimulation of de novo pyrimidine synthesis by growth signaling through mTOR and S6K1. Science 2013; 339: 1323–1328. 10.1126/science.1228792 PMC3753690 23429703

[bib7] 7Hardie DG. AMPK: positive and negative regulation, and its role in whole-body energy homeostasis. Curr Opin Cell Biol 2015; 33: 1–7. 25259783 10.1016/j.ceb.2014.09.004

[bib8] 8Jenne DE, Reimann H, Nezu J, Friedel W, Loff S, Jeschke R et al. Peutz-Jeghers syndrome is caused by mutations in a novel serine threonine kinase. Nat Genet 1998; 18: 38–43. 9425897 10.1038/ng0198-38

[bib9] 9Xie M, Zhang D, Dyck JR, Li Y, Zhang H, Morishima M et al. A pivotal role for endogenous TGF-beta-activated kinase-1 in the LKB1/AMP-activated protein kinase energy-sensor pathway. Proc Natl Acad Sci USA 2006; 103: 17378–17383. 17085580 10.1073/pnas.0604708103 PMC1859937

[bib10] 10Foretz M, Guigas B, Bertrand L, Pollak M, Viollet B. Metformin: from mechanisms of action to therapies. Cell Metab 2014; 20: 953–966. 25456737 10.1016/j.cmet.2014.09.018

[bib11] 11Zhou G, Myers R, Li Y, Chen Y, Shen X, Fenyk-Melody J et al. Role of AMP-activated protein kinase in mechanism of metformin action. J Clin Invest 2001; 108: 1167–1174. 11602624 10.1172/JCI13505 PMC209533

[bib12] 12Hawley SA, Boudeau J, Reid JL, Mustard KJ, Udd L, Makela TP et al. Complexes between the LKB1 tumor suppressor, STRAD alpha/beta and MO25 alpha/beta are upstream kinases in the AMP-activated protein kinase cascade. J Biol 2003; 2: 28. 14511394 10.1186/1475-4924-2-28 PMC333410

[bib13] 13Evans JM, Donnelly LA, Emslie-Smith AM, Alessi DR, Morris AD. Metformin and reduced risk of cancer in diabetic patients. BMJ 2005; 330: 1304–1305. 15849206 10.1136/bmj.38415.708634.F7 PMC558205

[bib14] 14Suissa S, Azoulay L. Metformin and cancer: mounting evidence against an association. Diabetes Care 2014; 37: 1786–1788. 24963109 10.2337/dc14-0500

[bib15] 15Ben Sahra I, Laurent K, Loubat A, Giorgetti-Peraldi S, Colosetti P, Auberger P et al. The antidiabetic drug metformin exerts an antitumoral effect in vitro and in vivo through a decrease of cyclin D1 level. Oncogene 2008; 27: 3576–3586. 18212742 10.1038/sj.onc.1211024

[bib16] 16Zakikhani M, Dowling R, Fantus IG, Sonenberg N, Pollak M. Metformin is an AMP kinase-dependent growth inhibitor for breast cancer cells. Cancer Res 2006; 66: 10269–10273. 17062558 10.1158/0008-5472.CAN-06-1500

[bib17] 17Dirat B, Ader I, Golzio M, Massa F, Mettouchi A, Laurent K et al. Inhibition of the GTPase Rac1 mediates the antimigratory effects of metformin in prostate cancer cells. Mol Cancer Ther 2015; 14: 586–596. 25527635 10.1158/1535-7163.MCT-14-0102

[bib18] 18Kisfalvi K, Eibl G, Sinnett-Smith J, Rozengurt E. Metformin disrupts crosstalk between G protein-coupled receptor and insulin receptor signaling systems and inhibits pancreatic cancer growth. Cancer Res 2009; 69: 6539–6545. 19679549 10.1158/0008-5472.CAN-09-0418 PMC2753241

[bib19] 19Anisimov VN, Berstein LM, Egormin PA, Piskunova TS, Popovich IG, Zabezhinski MA et al. Effect of metformin on life span and on the development of spontaneous mammary tumors in HER-2/neu transgenic mice. Exp Gerontol 2005; 40: 685–693. 16125352 10.1016/j.exger.2005.07.007

[bib20] 20Huang X, Wullschleger S, Shpiro N, McGuire VA, Sakamoto K, Woods YL et al. Important role of the LKB1-AMPK pathway in suppressing tumorigenesis in PTEN-deficient mice. Biochem J 2008; 412: 211–221. 18387000 10.1042/BJ20080557

[bib21] 21Cerezo M, Tichet M, Abbe P, Ohanna M, Lehraiki A, Rouaud F et al. Metformin blocks melanoma invasion and metastasis development in a AMPK/p53-dependent manner. Mol Cancer Ther 2013; 12: 1605–1615. 23741061 10.1158/1535-7163.MCT-12-1226-T

[bib22] 22Buzzai M, Jones RG, Amaravadi RK, Lum JJ, DeBerardinis RJ, Zhao F et al. Systemic treatment with the antidiabetic drug metformin selectively impairs p53-deficient tumor cell growth. Cancer Res 2007; 67: 6745–6752. 17638885 10.1158/0008-5472.CAN-06-4447

[bib23] 23Tomic T, Botton T, Cerezo M, Robert G, Luciano F, Puissant A et al. Metformin inhibits melanoma development through autophagy and apoptosis mechanisms. Cell Death Dis 2011; 2: e199. 21881601 10.1038/cddis.2011.86 PMC3186904

[bib24] 24El-Mir MY, Nogueira V, Fontaine E, Averet N, Rigoulet M, Leverve X. Dimethylbiguanide inhibits cell respiration via an indirect effect targeted on the respiratory chain complex I. J Biol Chem 2000; 275: 223–228. 10617608 10.1074/jbc.275.1.223

[bib25] 25Owen MR, Doran E, Halestrap AP. Evidence that metformin exerts its anti-diabetic effects through inhibition of complex 1 of the mitochondrial respiratory chain. Biochem J 2000; 348(Pt 3): 607–614. 10839993 PMC1221104

[bib26] 26Fontaine E. Metformin and respiratory chain complex I: the last piece of the puzzle? Biochem J 2014; 463: e3–e5. 25301073 10.1042/BJ20141020

[bib27] 27Bridges HR, Jones AJ, Pollak MN, Hirst J. Effects of metformin and other biguanides on oxidative phosphorylation in mitochondria. Biochem J 2014; 462: 475–487. 25017630 10.1042/BJ20140620 PMC4148174

[bib28] 28Ben Sahra I, Laurent K, Giuliano S, Larbret F, Ponzio G, Gounon P et al. Targeting cancer cell metabolism: the combination of metformin and 2-deoxyglucose induces p53-dependent apoptosis in prostate cancer cells. Cancer Res 2010; 70: 2465–2475. 20215500 10.1158/0008-5472.CAN-09-2782

[bib29] 29Wheaton WW, Weinberg SE, Hamanaka RB, Soberanes S, Sullivan LB, Anso E et al. Metformin inhibits mitochondrial complex I of cancer cells to reduce tumorigenesis. Elife 2014; 3: e02242. 24843020 10.7554/eLife.02242 PMC4017650

[bib30] 30Foretz M, Hebrard S, Leclerc J, Zarrinpashneh E, Soty M, Mithieux G et al. Metformin inhibits hepatic gluconeogenesis in mice independently of the LKB1/AMPK pathway via a decrease in hepatic energy state. J Clin Invest 2010; 120: 2355–2369. 20577053 10.1172/JCI40671 PMC2898585

[bib31] 31Loubiere C, Goiran T, Laurent K, Djabari Z, Tanti JF, Bost F. Metformin-induced energy deficiency leads to the inhibition of lipogenesis in prostate cancer cells. Oncotarget 2015; 6: 15652–15661. 26002551 10.18632/oncotarget.3404 PMC4558177

[bib32] 32Fendt SM, Bell EL, Keibler MA, Davidson SM, Wirth GJ, Fiske B et al. Metformin decreases glucose oxidation and increases the dependency of prostate cancer cells on reductive glutamine metabolism. Cancer Res 2013; 73: 4429–4438. 23687346 10.1158/0008-5472.CAN-13-0080 PMC3930683

[bib33] 33Hirsch HA, Iliopoulos D, Tsichlis PN, Struhl K. Metformin selectively targets cancer stem cells, and acts together with chemotherapy to block tumor growth and prolong remission. Cancer Res 2009; 69: 7507–7511. 19752085 10.1158/0008-5472.CAN-09-2994 PMC2756324

[bib34] 34Iliopoulos D, Hirsch HA, Struhl K. Metformin decreases the dose of chemotherapy for prolonging tumor remission in mouse xenografts involving multiple cancer cell types. Cancer Res 2010; 71: 3196–3201. 10.1158/0008-5472.CAN-10-3471 PMC3085572 21415163

[bib35] 35Bao B, Wang Z, Ali S, Ahmad A, Azmi AS, Sarkar SH et al. Metformin inhibits cell proliferation, migration and invasion by attenuating CSC function mediated by deregulating miRNAs in pancreatic cancer cells. Cancer Prev Res (Phila) 2012; 5: 355–364. 22086681 10.1158/1940-6207.CAPR-11-0299 PMC3786260

[bib36] 36Shank JJ, Yang K, Ghannam J, Cabrera L, Johnston CJ, Reynolds RK et al. Metformin targets ovarian cancer stem cells in vitro and in vivo. Gynecol Oncol 2012; 127: 390–397. 22864111 10.1016/j.ygyno.2012.07.115 PMC3580263

[bib37] 37Vazquez-Martin A, Oliveras-Ferraros C, Cufi S, Del Barco S, Martin-Castillo B, Menendez JA. Metformin regulates breast cancer stem cell ontogeny by transcriptional regulation of the epithelial-mesenchymal transition (EMT) status. Cell Cycle 2010; 9: 3807–3814. 20890129

[bib38] 38Sancho P, Burgos-Ramos E, Tavera A, Bou Kheir T, Jagust P, Schoenhals M et al. MYC/PGC-1alpha balance determines the metabolic phenotype and plasticity of pancreatic cancer stem cells. Cell Metab 2015; 22: 590–605. 26365176 10.1016/j.cmet.2015.08.015

[bib39] 39Gotlieb WH, Saumet J, Beauchamp MC, Gu J, Lau S, Pollak MN et al. In vitro metformin anti-neoplastic activity in epithelial ovarian cancer. Gynecol Oncol 2008; 19: 19. 10.1016/j.ygyno.2008.04.008 18495226

[bib40] 40Dowling RJ, Zakikhani M, Fantus IG, Pollak M, Sonenberg N. Metformin inhibits mammalian target of rapamycin-dependent translation initiation in breast cancer cells. Cancer Res 2007; 67: 10804–10812. 18006825 10.1158/0008-5472.CAN-07-2310

[bib41] 41Ben Sahra I, Regazzetti C, Robert G, Laurent K, Le Marchand-Brustel Y, Auberger P et al. Metformin, independent of AMPK, induces mTOR inhibition and cell-cycle arrest through REDD1. Cancer Res 2011; 71: 4366–4372. 21540236 10.1158/0008-5472.CAN-10-1769

[bib42] 42Kalender A, Selvaraj A, Kim SY, Gulati P, Brule S, Viollet B et al. Metformin, independent of AMPK, inhibits mTORC1 in a rag GTPase-dependent manner. Cell Metab 2010; 11: 390–401. 20444419 10.1016/j.cmet.2010.03.014 PMC3081779

[bib43] 43Possemato R, Marks KM, Shaul YD, Pacold ME, Kim D, Birsoy K et al. Functional genomics reveal that the serine synthesis pathway is essential in breast cancer. Nature 2011; 476: 346–350. 21760589 10.1038/nature10350 PMC3353325

[bib44] 44Pollak N, Dolle C, Ziegler M. The power to reduce: pyridine nucleotides—small molecules with a multitude of functions. Biochem J 2007; 402: 205–218. 17295611 10.1042/BJ20061638 PMC1798440

[bib45] 45Scarbrough PM, Mapuskar KA, Mattson DM, Gius D, Watson WH, Spitz DR. Simultaneous inhibition of glutathione- and thioredoxin-dependent metabolism is necessary to potentiate 17AAG-induced cancer cell killing via oxidative stress. Free Radic Biol Med 2012; 52: 436–443. 22100505 10.1016/j.freeradbiomed.2011.10.493 PMC3664944

[bib46] 46Boutros J, Almasan A. Combining 2-deoxy-D-glucose with electron transport chain blockers: a double-edged sword. Cancer Biol Ther 2009; 8: 1237–1238. 19458493 10.4161/cbt.8.13.8869 PMC2923584

[bib47] 47Schachter H. Complex N-glycans: the story of the ‘yellow brick road'. Glycoconj J 2014; 31: 1–5. 24178944 10.1007/s10719-013-9507-5

[bib48] 48Qin JZ, Xin H, Nickoloff BJ. 2-deoxyglucose sensitizes melanoma cells to TRAIL-induced apoptosis which is reduced by mannose. Biochem Biophys Res Commun 2010; 401: 293–299. 20851102 10.1016/j.bbrc.2010.09.054

[bib49] 49Ramirez-Peinado S, Alcazar-Limones F, Lagares-Tena L, El Mjiyad N, Caro-Maldonado A, Tirado OM et al. 2-deoxyglucose induces Noxa-dependent apoptosis in alveolar rhabdomyosarcoma. Cancer Res 2011; 71: 6796–6806. 21911456 10.1158/0008-5472.CAN-11-0759

[bib50] 50Zhu Z, Jiang W, McGinley JN, Thompson HJ. 2-Deoxyglucose as an energy restriction mimetic agent: effects on mammary carcinogenesis and on mammary tumor cell growth in vitro. Cancer Res 2005; 65: 7023–7030. 16061689 10.1158/0008-5472.CAN-05-0453

[bib51] 51Ben-Sahra I, Dirat B, Laurent K, Puissant A, Auberger P, Budanov A et al. Sestrin2 integrates Akt and mTOR signaling to protect cells against energetic stress-induced death. Cell Death Differ 2013; 20: 611–619. 23238567 10.1038/cdd.2012.157 PMC3595485

[bib52] 52Corton JM, Gillespie JG, Hawley SA, Hardie DG. 5-aminoimidazole-4-carboxamide ribonucleoside A specific method for activating AMP-activated protein kinase in intact cells? Eur J Biochem 1995; 229: 558–565. 7744080 10.1111/j.1432-1033.1995.tb20498.x

[bib53] 53Narkar VA, Downes M, Yu RT, Embler E, Wang YX, Banayo E et al. AMPK and PPARdelta agonists are exercise mimetics. Cell 2008; 134: 405–415. 18674809 10.1016/j.cell.2008.06.051 PMC2706130

[bib54] 54Niederberger E, King TS, Russe OQ, Geisslinger G. Activation of AMPK and its impact on exercise capacity. Sports Med 2015; 45: 1497–1509. 26186961 10.1007/s40279-015-0366-z

[bib55] 55Henin N, Vincent MF, Gruber HE, Van den Berghe G. Inhibition of fatty acid and cholesterol synthesis by stimulation of AMP-activated protein kinase. FASEB J 1995; 9: 541–546. 7737463 10.1096/fasebj.9.7.7737463

[bib56] 56Horman S, Browne G, Krause U, Patel J, Vertommen D, Bertrand L et al. Activation of AMP-activated protein kinase leads to the phosphorylation of elongation factor 2 and an inhibition of protein synthesis. Curr Biol 2002; 12: 1419–1423. 12194824 10.1016/s0960-9822(02)01077-1

[bib57] 57Koistinen HA, Galuska D, Chibalin AV, Yang J, Zierath JR, Holman GD et al. 5-Amino-imidazole carboxamide riboside increases glucose transport and cell-surface GLUT4 content in skeletal muscle from subjects with type 2 diabetes. Diabetes 2003; 52: 1066–1072. 12716734 10.2337/diabetes.52.5.1066

[bib58] 58Pold R, Jensen LS, Jessen N, Buhl ES, Schmitz O, Flyvbjerg A et al. Long-term AICAR administration and exercise prevents diabetes in ZDF rats. Diabetes 2005; 54: 928–934. 15793229 10.2337/diabetes.54.4.928

[bib59] 59Babraj JA, Mustard K, Sutherland C, Towler MC, Chen S, Smith K et al. Blunting of AICAR-induced human skeletal muscle glucose uptake in type 2 diabetes is dependent on age rather than diabetic status. Am J Physiol Endocrinol Metab 2009; 296: E1042–E1048. 19190259 10.1152/ajpendo.90811.2008 PMC2681307

[bib60] 60Boon H, Bosselaar M, Praet SF, Blaak EE, Saris WH, Wagenmakers AJ et al. Intravenous AICAR administration reduces hepatic glucose output and inhibits whole body lipolysis in type 2 diabetic patients. Diabetologia 2008; 51: 1893–1900. 18709353 10.1007/s00125-008-1108-7

[bib61] 61Cuthbertson DJ, Babraj JA, Mustard KJ, Towler MC, Green KA, Wackerhage H et al. 5-Aminoimidazole-4-carboxamide 1-beta-D-ribofuranoside acutely stimulates skeletal muscle 2-deoxyglucose uptake in healthy men. Diabetes 2007; 56: 2078–2084. 17513706 10.2337/db06-1716

[bib62] 62Campas C, Lopez JM, Santidrian AF, Barragan M, Bellosillo B, Colomer D et al. Acadesine activates AMPK and induces apoptosis in B-cell chronic lymphocytic leukemia cells but not in T lymphocytes. Blood 2003; 101: 3674–3680. 12522004 10.1182/blood-2002-07-2339

[bib63] 63Nafz J, De-Castro Arce J, Fleig V, Patzelt A, Mazurek S, Rosl F. Interference with energy metabolism by 5-aminoimidazole-4-carboxamide-1-beta-D-ribofuranoside induces HPV suppression in cervical carcinoma cells and apoptosis in the absence of LKB1. Biochem J 2007; 403: 501–510. 17212587 10.1042/BJ20061053 PMC1876364

[bib64] 64Rattan R, Giri S, Singh AK, Singh I. 5-Aminoimidazole-4-carboxamide-1-beta-D-ribofuranoside inhibits cancer cell proliferation in vitro and in vivo via AMP-activated protein kinase. J Biol Chem 2005; 280: 39582–39593. 16176927 10.1074/jbc.M507443200

[bib65] 65Robert G, Ben Sahra I, Puissant A, Colosetti P, Belhacene N, Gounon P et al. Acadesine kills chronic myelogenous leukemia (CML) cells through PKC-dependent induction of autophagic cell death. PLoS ONE 2009; 4: e7889. 19924252 10.1371/journal.pone.0007889 PMC2775681

[bib66] 66Sengupta TK, Leclerc GM, Hsieh-Kinser TT, Leclerc GJ, Singh I, Barredo JC. Cytotoxic effect of 5-aminoimidazole-4-carboxamide-1-beta-4-ribofuranoside (AICAR) on childhood acute lymphoblastic leukemia (ALL) cells: implication for targeted therapy. Mol Cancer 2007; 6: 46. 17623090 10.1186/1476-4598-6-46 PMC1948012

[bib67] 67Santidrian AF, Gonzalez-Girones DM, Iglesias-Serret D, Coll-Mulet L, Cosialls AM, de Frias M et al. AICAR induces apoptosis independently of AMPK and p53 through up-regulation of the BH3-only proteins BIM and NOXA in chronic lymphocytic leukemia cells. Blood 2010; 116: 3023–3032. 20664053 10.1182/blood-2010-05-283960

[bib68] 68Montraveta A, Xargay-Torrent S, Lopez-Guerra M, Rosich L, Perez-Galan P, Salaverria I et al. Synergistic anti-tumor activity of acadesine (AICAR) in combination with the anti-CD20 monoclonal antibody rituximab in in vivo and in vitro models of mantle cell lymphoma. Oncotarget 2014; 5: 726–739. 24519895 10.18632/oncotarget.1455 PMC3996675

[bib69] 69Vakana E, Altman JK, Glaser H, Donato NJ, Platanias LC. Antileukemic effects of AMPK activators on BCR-ABL-expressing cells. Blood 2011; 118: 6399–6402. 22021366 10.1182/blood-2011-01-332783 PMC3236122

[bib70] 70Rosilio C, Lounnas N, Nebout M, Imbert V, Hagenbeek T, Spits H et al. The metabolic perturbators metformin, phenformin and AICAR interfere with the growth and survival of murine PTEN-deficient T cell lymphomas and human T-ALL/T-LL cancer cells. Cancer Lett 2013; 336: 114–126. 23612073 10.1016/j.canlet.2013.04.015

[bib71] 71Chuang HC, Chou CC, Kulp SK, Chen CS. AMPK as a potential anticancer target - friend or foe? Curr Pharm Des 2014; 20: 2607–2618. 23859619 10.2174/13816128113199990485 PMC4264967

[bib72] 72Jeon SM, Chandel NS, Hay N. AMPK regulates NADPH homeostasis to promote tumour cell survival during energy stress. Nature 2012; 485: 661–665. 22660331 10.1038/nature11066 PMC3607316

[bib73] 73Kato K, Ogura T, Kishimoto A, Minegishi Y, Nakajima N, Miyazaki M et al. Critical roles of AMP-activated protein kinase in constitutive tolerance of cancer cells to nutrient deprivation and tumor formation. Oncogene 2002; 21: 6082–6090. 12203120 10.1038/sj.onc.1205737

[bib74] 74Park HU, Suy S, Danner M, Dailey V, Zhang Y, Li H et al. AMP-activated protein kinase promotes human prostate cancer cell growth and survival. Mol Cancer Ther 2009; 8: 733–741. 19372545 10.1158/1535-7163.MCT-08-0631 PMC2775041

[bib75] 75Hosono K, Endo H, Takahashi H, Sugiyama M, Sakai E, Uchiyama T et al. Metformin suppresses colorectal aberrant crypt foci in a short-term clinical trial. Cancer Prev Res (Phila Pa) 2010; 3: 1077–1083. 10.1158/1940-6207.CAPR-10-0186 20810669

[bib76] 76Joshua AM, Zannella VE, Downes MR, Bowes B, Hersey K, Koritzinsky M et al. A pilot 'window of opportunity' neoadjuvant study of metformin in localised prostate cancer. Prostate Cancer Prostatic Dis 2014; 17: 252–258. 24861559 10.1038/pcan.2014.20

[bib77] 77Kordes S, Pollak MN, Zwinderman AH, Mathot RA, Weterman MJ, Beeker A et al. Metformin in patients with advanced pancreatic cancer: a double-blind, randomised, placebo-controlled phase 2 trial. Lancet Oncol 2015; 16: 839–847. 26067687 10.1016/S1470-2045(15)00027-3

[bib78] 78Landau BR, Laszlo J, Stengle J, Burk D. Certain metabolic and pharmacologic effects in cancer patients given infusions of 2-deoxy-D-glucose. J Natl Cancer Inst 1958; 21: 485–494. 13576102

[bib79] 79Mohanti BK, Rath GK, Anantha N, Kannan V, Das BS, Chandramouli BA et al. Improving cancer radiotherapy with 2-deoxy-D-glucose: phase I/II clinical trials on human cerebral gliomas. Int J Radiat Oncol Biol Phys 1996; 35: 103–111. 8641905 10.1016/s0360-3016(96)85017-6

[bib80] 80Raez LE, Papadopoulos K, Ricart AD, Chiorean EG, Dipaola RS, Stein MN et al. A phase I dose-escalation trial of 2-deoxy-D-glucose alone or combined with docetaxel in patients with advanced solid tumors. Cancer Chemother Pharmacol 2013; 71: 523–530. 23228990 10.1007/s00280-012-2045-1

[bib81] 81Stein M, Lin H, Jeyamohan C, Dvorzhinski D, Gounder M, Bray K et al. Targeting tumor metabolism with 2-deoxyglucose in patients with castrate-resistant prostate cancer and advanced malignancies. Prostate 2010; 70: 1388–1394. 20687211 10.1002/pros.21172 PMC4142700

[bib82] 82Mullane K. Acadesine: the prototype adenosine regulating agent for reducing myocardial ischaemic injury. Cardiovasc Res 1993; 27: 43–47. 8458030 10.1093/cvr/27.1.43

[bib83] 83Van Den Neste E, Cazin B, Janssens A, Gonzalez-Barca E, Terol MJ, Levy V et al. Acadesine for patients with relapsed/refractory chronic lymphocytic leukemia (CLL): a multicenter phase I/II study. Cancer Chemother Pharmacol 2013; 71: 581–591. 23228986 10.1007/s00280-012-2033-5 PMC3579463

[bib84] 84Ben Sahra I, Tanti JF, Bost F. The combination of metformin and 2-deoxyglucose inhibits autophagy and induces AMPK dependent apoptosis in prostate cancer cells. Autophagy 2010; 6: 670. 20559023 10.4161/auto.6.5.12434

[bib85] 85Cheong JH, Park ES, Liang J, Dennison JB, Tsavachidou D, Nguyen-Charles C et al. Dual inhibition of tumor energy pathway by 2-deoxyglucose and metformin is effective against a broad spectrum of preclinical cancer models. Mol Cancer Ther 2011; 10: 2350–2362. 21992792 10.1158/1535-7163.MCT-11-0497 PMC3237863

[bib86] 86Shu Y, Sheardown SA, Brown C, Owen RP, Zhang S, Castro RA et al. Effect of genetic variation in the organic cation transporter 1 (OCT1) on metformin action. J Clin Invest 2007; 117: 1422–1431. 17476361 10.1172/JCI30558 PMC1857259

[bib87] 87Birsoy K, Possemato R, Lorbeer FK, Bayraktar EC, Thiru P, Yucel B et al. Metabolic determinants of cancer cell sensitivity to glucose limitation and biguanides. Nature 2014; 508: 108–112. 24670634 10.1038/nature13110 PMC4012432

[bib88] 88Beneteau M, Zunino B, Jacquin MA, Meynet O, Chiche J, Pradelli LA et al. Combination of glycolysis inhibition with chemotherapy results in an antitumor immune response. Proc Natl Acad Sci USA 2012; 109: 20071–20076. 23169636 10.1073/pnas.1206360109 PMC3523878

[bib89] 89Eikawa S, Nishida M, Mizukami S, Yamazaki C, Nakayama E, Udono H. Immune-mediated antitumor effect by type 2 diabetes drug, metformin. Proc Natl Acad Sci USA 2015; 112: 1809–1814. 25624476 10.1073/pnas.1417636112 PMC4330733

